# Dissection of Recombination Attributes for Multiple Maize Populations Using a Common SNP Assay

**DOI:** 10.3389/fpls.2017.02063

**Published:** 2017-11-30

**Authors:** Haiying Guan, Farhan Ali, Qingchun Pan

**Affiliations:** ^1^Maize Research Institute, Shandong Academy of Agricultural Sciences, Jinan, China; ^2^National Engineering Laboratory of Wheat and Maize, Jinan, China; ^3^Key Laboratory of Biology and Genetic Improvement of Maize in Northern Yellow-Huai River Plain, Ministry of Agriculture, Jinan, China; ^4^Cereal Crops Research Institute, Nowshera, Pakistan; ^5^National Key Laboratory of Crop Genetic Improvement, Huazhong Agricultural University, Wuhan, China

**Keywords:** maize, SNP, recombination event, recombination bin, recombination frequency

## Abstract

Recombination is a vital characteristic for quantitative trait loci mapping and breeding to enhance the yield potential of maize. However, recombination characteristics in globally used segregating populations have never been evaluated at similar genetic marker densities. This study aimed to divulge the characteristics of recombination events, recombinant chromosomal segments, and recombination frequency for four dissimilar populations. These populations were doubled haploid (DH), recombination inbred line (RIL), intermated B73xMo17 (IBM), and multi-parent advanced generation inter-cross (MAGIC), using the Illumina MaizeSNP50 BeadChip to provide markers. Our results revealed that the average number of recombination events was 16, 41, 72, and 86 per line in DH, RIL, IBM, and MAGIC populations, respectively. Accordingly, the average length of recombinant chromosomal segments was 84.8, 47.3, 29.2, and 20.4 Mb in DH, RIL, IBM, and MAGIC populations, respectively. Furtherly, the recombination frequency varied in different genomic regions and population types [DH (0–12.7 cM/Mb), RIL (0–15.5 cM/Mb), IBM (0–24.1 cM/Mb), MAGIC (0–42.3 cM/Mb)]. Utilizing different sub-sets of lines, the recombination bin number and size were analyzed in each population. Additionally, different sub-sets of markers and lines were employed to estimate the recombination bin number and size via formulas for relationship in these populations. The relationship between recombination events and recombination bin length was also examined. Our results contribute to determining the most suitable number of genetic markers, lines in each population, and population type for successful mapping and breeding.

## Introduction

During the processes of synapsis and crossing-over new combination of alleles occurs by reciprocal exchanges of genetic material between homologous chromosomes. The resultant offspring can play an important role in the process of genomic evolution and formation of genetic diversity (Zhang and Gaut, 2003; Meunier and Duret, 2004; Gaut et al., 2007). The process of recombination is of great importance to crop improvement by breeding, and also facilitates gene mapping and utilization of new techniques to accelerate breeding progress (Kromdijk et al., 2014). Recombination is one of several bottlenecks that need to be addressed in a successful, target-oriented breeding program. Comparison of the recombination characteristics of different types of population to guide the process of QTL mapping has been studied little. This study was performed to facilitate future breeding programs, mapping target QTL_S_/genes at minimal cost and by the easiest method.

Several economically and genetically important plant species have reference genome sequences^1^. Maize is one of the most important crops for genetic study because of its complex genomic attributes. The B73 reference genome was published in 2009 (Schnable et al., 2009). Subsequently, several lines were re-sequenced to identify and characterize high-density stable single-nucleotide polymorphisms (SNPs) (Gore et al., 2009a,b; Chia et al., 2012). The abundance of markers and accessibility of different genetic sources offer potential solutions to many genetic impediment and breeding bottlenecks (Yu et al., 2014). Recently, different sub-sets of SNP assays were developed for application in species evolution, QTL/gene mapping, genomic selection, and marker-assisted breeding (Yan et al., 2009; Lu et al., 2010; Pan et al., 2012; Leung et al., 2015; Liu L. et al., 2015; Singh et al., 2015). Recombination frequency with high-density markers’ estimation taken as the important recombination characteristics was also popularly used in different trait mapping and heterosis prediction (Xu, 2013; Chen et al., 2014; Li C. et al., 2015; Zhou et al., 2016; Su et al., 2017). Recombination frequency comparison using three recombinant inbred line populations with the same middle density marker set was performed and variation was observed between different genomic regions and across three populations (Farkhari et al., 2011). The MaizeSNP50 assay was first reported in two intercross populations to compare the physical and genetic consistency of the B73 genome (Ganal et al., 2011). Subsequently, this SNP assay was utilized extensively, for example to explore several important economic traits and genetic attributes of maize via different analytical strategies including genome wide association study (GWAS) and linkage mapping (Weng et al., 2011; Bauer et al., 2013; Ding et al., 2015; Pan et al., 2016; Xiao et al., 2016). This set of markers was used for two different association mapping panels to assess germplasm diversity and explore genetic mechanisms underlying complex traits in maize (Weng et al., 2011; Yang et al., 2011). It was also used to reveal variation of inter-population recombination patterns among 23 doubled haploid (DH) populations, and to dissect the relationship of recombination with agronomic characteristics and gene expression in 11 recombinant inbred lines (RILs) and 1 BC_2_F_5_ population (Bauer et al., 2013; Pan et al., 2016). This set of DNA markers has been of widespread value in resolving obstacles to breeding progress (Liu L. et al., 2015).

Doubled haploid, RIL, intermated B73xMo17 (IBM), and multi-parent advanced generation inter-cross (MAGIC) populations are all suitable for QTL mapping as well as breeding because their offspring inherit balanced allele sets with exchanges from their parents. In *Arabidopsis*, a creative approach of centromere-mediated genome elimination was developed to construct DH populations; most haploids could be spontaneously doubled. The recombination rate was similar between DH and RIL populations (Seymour et al., 2012). RIL populations are widely used for mapping because they can be reproduced easily and more recombination events can be observed than in other populations. Production of RILs may consume much time due to the need for several generations of selfing, but the inputs are less compared with generating DH populations in most crops (Seymour et al., 2012). The IBM population was constructed with the aim of obtaining more recombination events in offspring and improving map resolution (Lee et al., 2002). It increases the number of recombination events relative to RILs by intercross pollination in early generations (Liu H. et al., 2015). In mice, *Arabidopsis*, rice, wheat, and maize, MAGIC populations were developed for gene mapping and generating new germplasm resources for breeding (Mott et al., 2000; Kover et al., 2009; Bandillo et al., 2013; Rebetzke et al., 2014). MAGIC populations increase mapping efficiency by evaluating more alleles at one locus and increasing recombination events in the offspring (Dell’Acqua et al., 2015).

In the present scenario, it is of prime importance to compare the important genetic patterns among these four different populations with a common set of markers. This study will provide a strong backdrop for future research to choose the most suitable population type, population size, and marker density for successful mapping and target-oriented breeding schemes.

## Materials and Methods

### Populations

Genetic material comprising DH, RIL, IBM, and MAGIC populations was utilized in this investigation. The process of developing each population is different as summarized (Supplementary Figure S1). Two diverse parental lines have been used for the development of each DH, RIL, and IBM population. The DH population was generated via haploid production, then doubling the chromosome number using colchicine. The RIL population was produced by crossing two parental lines to generate F_1_, with self-pollination to generate F_2_, then self-pollination of F_2_-derived lines for at least six generations. A unique feature of the IBM population made it distinct from the RIL population, in that the F_2_ was inter-mated randomly to generate F_3_ and the F_3_ random-mated to generate F_4_, then the F_4_ was self-pollinated to generate F_5_∼F_6_. The MAGIC population construction process was similar to IBM, except for use of multiple different parental lines. The F_1_ and F_2_ from different parental lines were randomly mated (Kover et al., 2009). The eight parents used in this study were divided into two sub-groups. The F_1_ was also divided into two sub-groups and the F_2_ was developed by crossing two F_1_s coming from different parents in the same sub-group. Subsequently, the F_3_ was developed by crossing two F_2_s from different sub-groups, and the F_3_ was self-pollinated to produce F_4_ to F_6_.

The number of parents and lines in each population was different, comprising a large dataset for comparing genetic characteristics in different populations (Supplementary Table S1). Among these four types of populations, the number of populations was 23, 11, 1, and 1 in DH, RIL, IBM, and MAGIC, respectively. A total of 2,233 lines were included in the 23 DH populations (Their parents were F353, UH007, B73, D06, D09, EC169, F252, F618, Mo17, UH250, UH304, W117, D152, EC49A, EP44, EZ5, F03802, F2, F283, F64, UH006, UH009, and DK105, most of which were inbred lines in Europe.) and 2,218 lines were included in the 11 RIL families (Their parents were B73, BY804, BY815, BK, SK, ZONG3, YU87-1, DAN340, CI7, ZHENG58, K22, DE3, SC55, KUI3, and B77, most of which were elite inbred lines in China.), each of which was developed for exploring different complex traits via QTL mapping^2^. The IBM population (the parents were B73 and Mo17) consisted of 239 lines. Eight founders (A632, B73, B96, F7, H99, HP301, Mo17, and W153R) were used for generating the MAGIC population, and 303 lines were used in this study. Detailed information for these four populations was also provided and explained (Ganal et al., 2011; Bauer et al., 2013; Dell’Acqua et al., 2015; Pan et al., 2016).

The RIL used in this experiment is the property of Yan’s Lab^2^ and was described in detail previously (Pan et al., 2016). The DH, IBM, and MAGIC populations’ data were downloaded from three different articles, respectively (Ganal et al., 2011; Bauer et al., 2013; Dell’Acqua et al., 2015). These data were exploited for further analyses in order to determine the most appropriate population for recombination characteristic estimation and utility, and pinpoint the most suitable one for QTL mapping and breeding.

### Genotyping

The MaizeSNP50 Beadchip was used for genotyping these populations (Ganal et al., 2011; Bauer et al., 2013; Dell’Acqua et al., 2015; Pan et al., 2016). A total of 6,379–16,765; 11,360–15,285; 20,848; and 54,234 SNPs were polymorphic within each DH, RIL, IBM, and MAGIC population, respectively (Supplementary Table S1). All four data sets could be downloaded from the article supplementary files, respectively (Ganal et al., 2011; Bauer et al., 2013; Dell’Acqua et al., 2015; Pan et al., 2016). The original article markers whose missing rate less than 5% was reserved. Further, for each individual set, missing rate less than 5% was reserved.

### Estimation of Recombination Events, Chromosomal Segments, and Frequency

The MaizeSNP50 linkage map of B73 version 2 genome physical information was utilized to analyze the recombination patterns in DH, RIL, and IBM populations. Recombination events were counted as the number of recombination break points according to the linkage map and haplotypes originating from two or eight (MAGIC) parents. For the MAGIC population, the haplotype of family lines was constructed via the R package “happy” (Mott et al., 2000; Kover et al., 2009), with study of recombination patterns based on the completed haplotype of different lines using the B73 version 2 genome physical position of maize SNP50 markers. Because MAGIC family lines were from eight different parents, recombination events were calculated by the number of recombination break points from different parents contributing to the lines. For all populations, after determining the number of recombination break points, the length of recombinant chromosomal segments was calculated based on the distance between consecutive recombination break points. The frequency of recombination was assessed in all four populations by randomly selected 200 family lines and estimating recombination frequency variance in 2 Mb windows across the whole genome. The recombination frequency (cM/Mb) was calculated as the ratio of the number of recombinant lines to the total number of lines with 2 Mb physical length.

### Identification of Recombination Bin Number and Bin Size for Different Populations

A recombination bin was defined as a chromosomal segment devoid of recombination. Many lines were included in each segregating population, and the recombination bin number was examined by using more than one line, calculating the recombination bin number and size using the linkage map and haplotype markers. Different sub-sets of lines, 50, 100, 150, and 200 from DH, RIL, IBM, and MAGIC, respectively, were randomly selected. The mean value and standard deviation with 1,000 re-samples for bin number and size were measured.

### Prediction of Formulas between Recombination Bin Number and Size with Different Markers and Population Sets

Together with non-genetic factors that confer variation to phenotypes, the potential resolution of QTL mapping mainly relies on the recombination bin number, size, and population size. To estimate the map resolution of these four types of population, different sub-groups of markers were taken into account (200, 500, 1,000, 2,000, 5,000, 7,500, and 10,000) for each of the above-mentioned population sub-sets (50, 100, 150, and 200). Through this process, the varied numbers of markers and lines in different populations were utilized to reveal the recombination bin number and size. The formula for predicting bin number or bin size was as following:

$$\text{Y=m}\times {\text{X}}^{\text{n}}.$$

This formula was using the “nls” function in R, based on non-linear (weighted) least-squares estimates of the parameters with a non-linear model (Bates and Chambers, 1992), where *Y* is the recombination bin number or the length of recombination bin; *X* is the number of markers considered; and *m* and *n* are estimated coefficients of the formula.

### Estimation of Relationships between Recombination Events and Recombination Bin Number and Size

Maize is an extremely diverse species and possesses enormous genomic and phenotypic variability. However, the number of recombination events in each line of different populations is relatively stable. We combined the maize genome size, recombination events in each line and population size to estimate the theory of map resolution. We used the formula as following:

Y=a×X.

This formula was used to estimate the theoretical recombination bin number, where *Y* is the number of recombination bins; *a* is the mean number of recombination events of different populations, and *X* is the number of lines within the population. To test the theoretical recombination bin size and population size, we used the formula:

Y=2,300/(a×X),

where *Y* is the recombination bin size, in Mb, 2,300 is the maize genome length, in Mb, *a* is the average number of the recombination events in each line, and *X* is the number of lines in different populations.

### Simulations of Map Resolution in Different Population Types

In order to compare map resolution in four different population types, we randomly selected 200 lines combined with 1,000 normally distributed phenotype values. For DH, RIL, and IBM populations, we used the joint composite interval mapping (CIM) method in R/QTL (Broman et al., 2003). QTLs were identified based on likelihood of odds ratio (LOD) values above 3, with likelihood intervals defined by two LOD declines from the peak LOD. For MAGIC populations, we first constructed the linkage map then calculated the kinship between lines and performed linkage mapping by regressing phenotypes on the genotype probabilities produced by a Hidden Markov model (HMM) (Broman, 2005; Cheng et al., 2011, 2013). The 37th percentile was defined as the cut off for the 1,000 traits, with two LOD declines from the peak defined as the likelihood interval (Lander and Kruglyak, 1995). The MAGIC mapping method followed a previous article (Dell’Acqua et al., 2015).

## Results

### Variation in Numbers of Recombination Events, Lengths of Chromosomal Segments, and Local Recombination Frequency

A total of 23 DH, 11 RIL, 1 IBM, and 1 MAGIC population(s) with 2,233, 2,128, 239, and 303 lines, respectively, were genotyped via MaizeSNP50 chip high-density markers. Totals of 6,379–16,765, 11,360–15,285, 20,848, and 54,234 SNPs were polymorphic within DH, RIL, IBM, and MAGIC population(s), respectively (Supplementary Table S1). The number of recombination events in each population was detected according to the genetic map and haplotype. The greatest number of recombination events was observed in the RIL population (87,277), followed by the DH (34,741), MAGIC (26,058), and IBM (17,264; **Table 1**), although these values are confounded by large differences in population size. On average, 16 (ranging from 0 to 48), 41 (ranging from 16 to 104), 72 (ranging from 50 to 110), and 86 (ranging from 68 to 122) recombination events were observed per line in DH, RIL, IBM, and MAGIC population(s), respectively (Supplementary Figure S2 and **Table 1**). Variance of recombination event number among these populations was highly significant (one-way ANOVA; *F* = 3,203; *P* < 2.0E-16).

**Table 1:** Summary of recombination events for four types of population.

Pop. type	Number of families	Number of total recombination events	Mean ± sd^a^	Range of recombination events
DH	2,233	34,741	16 ± 12	0–48
RIL	2,128	87,277	41 ± 11	16–104
IBM	239	17,264	72 ± 11	50–110
MAGIC	303	26,058	86 ± 20	68–122

*^a^The average number of recombination events per line.*

**FIGURE 1:**
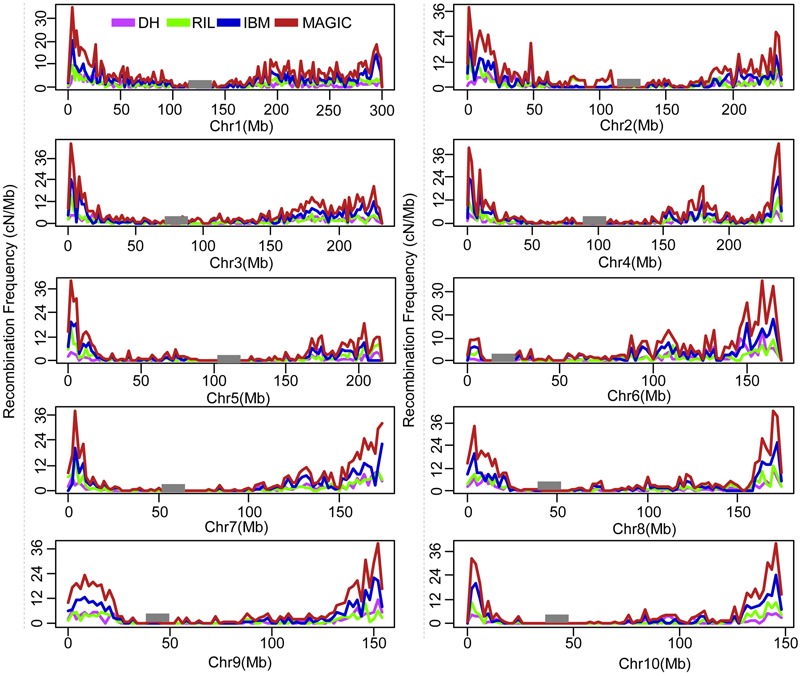
Genome wide of recombination frequency variance for four different population types. Recombination frequency of cM/Mb within 2 Mb window size was counted. Black rectangle is the centromeric position. DH, double haploid; RIL, recombination inbred lines; IBM, intermated B73xMo17; MAGIC, multi-parent advanced generation inter cross.

The recombination break points which divide each chromosome into different segments delineated the locations of recombination events. The length of non-recombinant chromosomal segments, calculated as the distance between consecutive recombination break points, averaged 84.8 (ranging from 2.0 to 301.1), 47.3 (ranging from 2.0 to 301.1), 29.2 (ranging from 1.0 to 260.2), and 20.4 (ranging from 0.78 to 230.5) Mb per line in DH, RIL, IBM, and MAGIC population(s), respectively (**Table 2**). Variance of recombination segment length was significant among these populations (one-way ANOVA; *F* = 7,797; *P* < 2.0E-16; Supplementary Figure S2). In order to compare the local recombination frequency variance, 200 lines were extracted randomly from the each of these four populations to analyze the recombination frequency variability with 2 Mb windows. There were significant differences across four populations between different chromosomes [DH (0–12.7 cM/Mb), RIL (0–15.5 cM/Mb), IBM (0–24.1 cM/Mb), MAGIC (0–42.3 cM/Mb); one-way ANOVA; *F* = 5.1E+33; *P* < 2.0E-16]. Further, the high recombination frequency mainly occurred in the non-centromeric region. Low recombination frequency was all found in the centromeric regions of the four groups (**Figure 1**).

**Table 2:** Summary of recombination segments in four types of population.

Pop. type	Number of families	Number of total recombination segments	Recombination segment length per line (Mb)^a^	Range of recombination segment lengths (Mb)
DH	2,233	51,290	84.8 ± 77.2	2.0–301.1
RIL	2,128	107,262	47.3 ± 50.3	2.0–301.1
IBM	239	19,533	29.2 ± 34.2	1.0–260.2
MAGIC	303	29,088	20.4 ± 27.5	0.78–230.5

*^a^The average length of recombination segments per line (Mb).*

### Variation in Recombination Bin Number and Size

**FIGURE 2:**
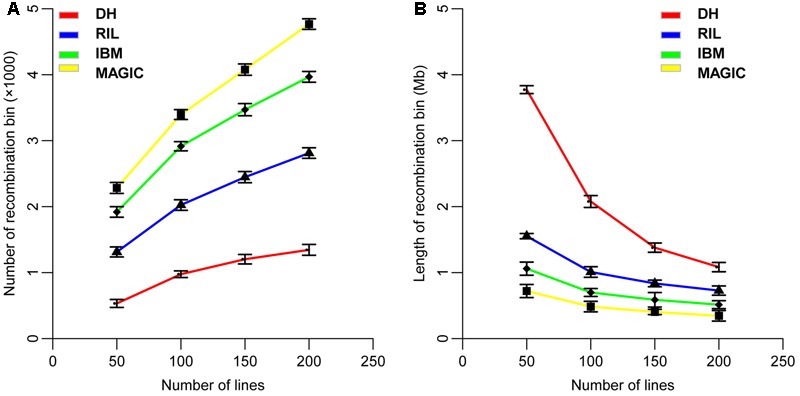
Distribution of the number and size of recombination bins in four types of populations. **(A)** Variation of recombination bin number with different sub-sets of line numbers in four populations. **(B)** Variation of recombination bin size with different sub-sets of lines in four populations. DH, double haploid; RIL, recombination inbred lines; IBM, intermated B73xMo17; MAGIC, multi-parent advanced generation inter cross.

The recombination bin number and size in each of these four populations were analyzed using high-density markers by dividing each population into sub-sets of 50, 100, 150, and 200 lines. The average result of 1,000 random re-samples was used to determine the average number of recombination bins as 533, 1,313, 1,919, and 2,283 in 50 lines; 976, 2,025, 2,915, and 3,396 in 100 lines; 1,203, 2,447, 3,469, and 4,077 in 150 lines; and 1,345, 2,812, 3,967, and 4,767 in 200 lines for DH, RIL, IBM, and MAGIC population(s), respectively (**Figure 2A** and **Table 3**). These results showed a direct relationship between the recombination bin number and the number of lines within each population. The recombination bin number varied significantly among different population types with the same number of lines (one-way ANOVA; *P* < 2.0E-16). The variance of recombination bin number with different random re-samples was stable, depicting that the recombination bin number of different sets of lines within the same type of population possessed less variance than did values for different populations.

**Table 3:** Variance of recombination bin number and size in four types of segregating population.

	Recombination bin number				Recombination bin size (Mb)			
Number of lines∖Pop. type	DH	RIL	IBM	MAGIC	DH	RIL	IBM	MAGIC
50	533	1,313	1,919	2,283	3.77	1.56	1.06	0.72
100	976	2,025	2,915	3,396	2.08	1.01	0.69	0.48
150	1,203	2,447	3,469	4,077	1.38	0.84	0.59	0.41
200	1,345	2,812	3,967	4,767	1.08	0.73	0.51	0.35

The recombination bin size was estimated to average 3.77, 1.56, 1.06, and 0.72 Mb in 50 lines; 2.08, 1.01, 0.69, and 0.48 Mb in 100 lines; 1.38, 0.84, 0.59, and 0.41 Mb in 150 lines; and 1.08, 0.73, 0.51, and 0.35 Mb in 200 lines for DH, RIL, IBM, and MAGIC population(s), respectively (**Figure 2B** and **Table 3**). Variation was observed for recombination bin number and size in all sub-groups within each population and even among these populations (**Figure 2**). Increasing the number of lines in each population increases the recombination bin number, but the increased level declined with the limited density of markers and recombination characteristics. However, the recombination bin length decreased with the increased number of lines in each population but decreasing levels gradually declined. These results depicted that the bin size is decreased in meager amounts with increasing numbers of lines in each population, which ultimately decreases the utility of extra-large populations. Therefore, a proper number of lines are required to maintain quality results at minimal cost and time.

### Estimation of Recombination Bin Number and Size with Different Sub-Sets of Markers and Lines

**FIGURE 3:**
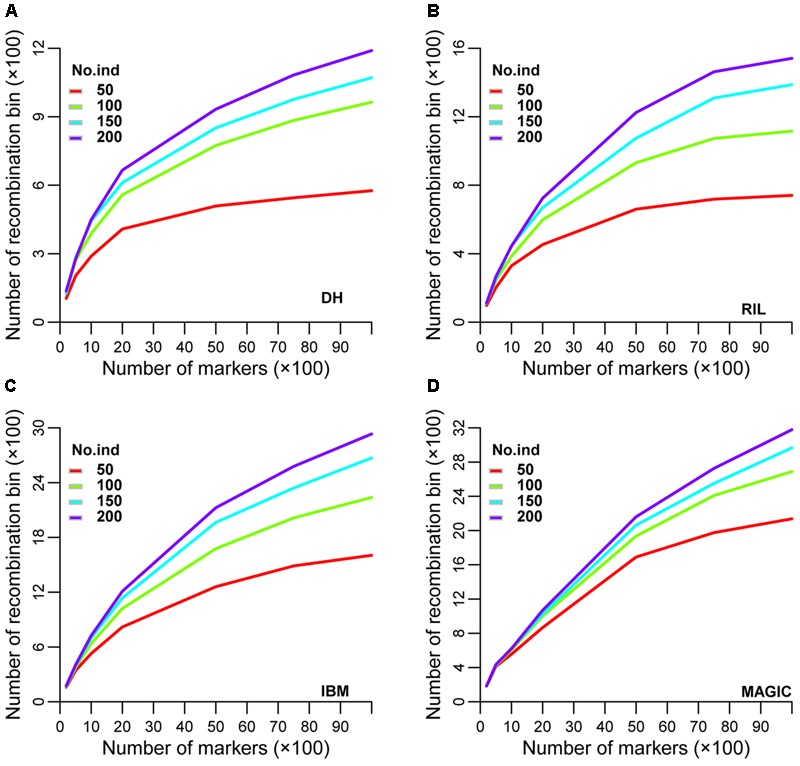
Simulation of recombination bin number in four types of population. Variation of recombination bin number among different densities of markers and numbers of lines in **(A)** DH, **(B)** RIL, **(C)** IBM, and **(D)** MAGIC population(s). DH, double haploid; RIL, recombination inbred lines; IBM, intermated B73xMo17; MAGIC, multi-parent advanced generation inter-cross.

Recombination bin number and size varied in different sub-sets of each population utilizing the same set of markers. An optimal set of markers and lines in each population is the corner stone to achieving genetics and breeding goals with minimal expenditure. To try to identify this optimum, a total of seven sub-groups of 200, 500, 1,000, 2,000, 5,000, 7,500, and 10,000 markers and four sub-sets of 50, 100, 150, and 200 lines in each population were examined for different genetic parameters. In the DH population, the range of recombination bin numbers was 104–1,190 among these seven sub-groups of markers and four sub-sets of lines (**Figure 3A** and Supplementary Table S2). The corresponding values in RIL, IBM, and MAGIC populations were 146–2,312, 160–2,933 and 182–3,179, respectively (**Figures 3B–D** and Supplementary Table S2). As expected, the number of recombination bins increased as the number of markers and lines increased. When marker number was fixed, the number of recombination bins increased less than the former datasets (that as marker and line number both increased) as line number increased. Among different sub-sets of lines with different marker sets in each type of population, the highest increased rate of recombination bin number was observed between 50 and 100 lines (**Figures 3A–D** and Supplementary Tables S2, S3). Among these populations the increase rate was lowest at all sub-sets in MAGIC. Additionally, the increasing rate (the increasing rate of recombination bin number was defined by this dataset divided the former dataset) of recombination bin number was most close to 1 when compared line sub-set 200–150 (Supplementary Table S3). These results showed that with smaller numbers of lines and markers, MAGIC had higher efficiency of separating closely linked loci by recombination. The most suitable (based on the increasing rate of recombination bin number was near 1) sub-set of lines was 200 for characterization via all sub-groups of markers (Supplementary Table S3).

**FIGURE 4:**
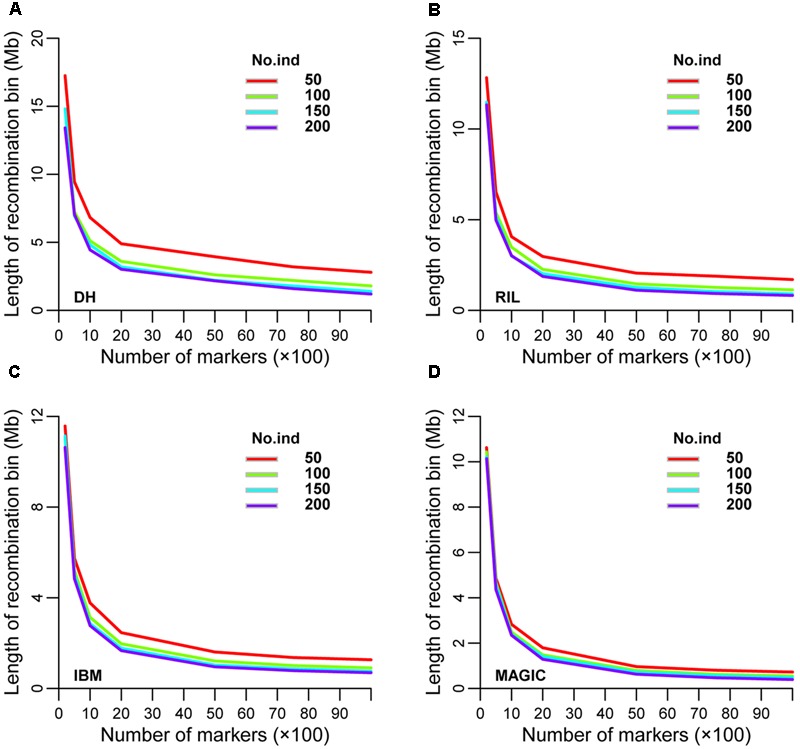
Prediction of recombination bin length in four types of population. Variation of recombination bin length among different densities of markers and numbers of lines in **(A)** DH, **(B)** RIL, **(C)** IBM, and **(D)** MAGIC population(s). DH, double haploid; RIL, recombination inbred lines; IBM, intermated B73xMo17; MAGIC, multi-parent advanced generation inter-cross.

Recombination bin size was also analyzed using the same seven sub-groups of markers and four sub-sets of lines used to evaluate bin number. The range of recombination bin sizes was 17.25–1.20, 12.83–0.82, 11.57–0.69, and 10.62–0.39 Mb among these sub-sets of markers and lines in DH, RIL, IBM, and MAGIC populations, respectively (**Figures 4A–D** and Supplementary Table S4). As expected, recombination bin length decreased as the number of markers and lines increased. When the marker number was fixed, recombination bin length decreased less than the former one dataset as line numbers increased. Comparing the recombination bin size in MAGIC, IBM, and RIL with DH population(s) for different marker sets showed that the sub-set of 5,000 markers provided preferential results regarding recombination bin size with full identification; moreover, these results were stable among different sub-sets of lines (Supplementary Table S5).

Increasing number and decreasing size of recombination bins with increased line and marker numbers in four types of population depicts the fixed inverse relationship between these parameters, despite the need for different formulas to estimate these parameters in each type of population (**Table 4**). As detailed below, to obtain a marker density that matches the average gene density in maize (1 per 57 Kb based on the genome size and a recent gene annotation), the required numbers of markers would be 970,453, 142,468, 121,844, and 47,001 in DH, RIL, IBM, and MAGIC populations, respectively.

**Table 4:** Formulas for recombination bin number and size for different sets of lines in four types of population.

Pop.	Lines	Bin size					Bin number				
		Formulas^a^	A1^b^	A2^c^	*P*-value^d^	*P*-value^e^	Formulas^f^	B1^g^	B2^h^	*P*-value^i^	*P*-value^j^
DH	50	*Y* = 250.04 ^∗^ *X*-0.51	27.1	0.018	2.93E-03	3.13E-05	*Y* = 26.60 ^∗^ *X*0.34	4.6	0.021	1.70E-02	3.96E-04
DH	100	*Y* = 354.58 ^∗^ *X*-0.61	46.7	0.023	6.36E-03	4.01E-05	*Y* = 21.05 ^∗^ *X*0.42	2.8	0.015	6.24E-03	3.59E-05
DH	150	*Y* = 495.47 ^∗^ *X*-0.67	61.7	0.022	5.09E-03	2.03E-05	*Y* = 21.74 ^∗^ *X*0.43	3.2	0.017	9.14E-03	5.34E-05
DH	200	*Y* = 390.24 ^∗^ *X*-0.64	29.5	0.013	5.94E-04	2.06E-06	*Y* = 19.39 ^∗^ *X*0.45	2.8	0.017	9.45E-03	4.20E-05
RIL	50	*Y* = 339.02 ^∗^ *X*-0.62	43.9	0.022	5.94E-03	3.27E-05	*Y* = 28.09 ^∗^ *X*0.41	5.5	0.023	2.58E-02	2.93E-04
RIL	100	*Y* = 418.64 ^∗^ *X*-0.69	43.6	0.018	2.44E-03	7.42E-06	*Y* = 21.53 ^∗^ *X*0.48	4.2	0.023	2.50E-02	1.21E-04
RIL	150	*Y* = 688.40 ^∗^ *X*-0.78	80.9	0.021	4.02E-03	8.01E-06	*Y* = 17.66 ^∗^ *X*0.52	3.1	0.020	1.72E-02	4.43E-05
RIL	200	*Y* = 763.21 ^∗^ *X*-0.80	81.6	0.019	2.72E-03	4.44E-06	*Y* = 16.26 ^∗^ *X*0.55	3.4	0.024	3.04E-02	8.60E-05
IBM	50	*Y* = 375.10 ^∗^ *X*-0.66	36.6	0.017	1.85E-03	6.38E-06	*Y* = 17.85 ^∗^*X* 0.49	2.6	0.017	9.75E-03	2.74E-05
IBM	100	*Y* = 572.11 ^∗^ *X*-0.75	51.4	0.016	1.29E-03	2.50E-06	*Y* = 14.35 ^∗^*X* 0.55	2.2	0.017	1.04E-02	1.66E-05
IBM	150	*Y* = 791.56 ^∗^ *X*-0.81	70.7	0.016	1.26E-03	1.74E-06	*Y* = 12.62 ^∗^ *X*0.58	1.9	0.018	1.15E-02	1.42E-05
IBM	200	*Y* = 757.13 ^∗^ *X*-0.81	56.4	0.013	5.54E-04	7.00E-07	*Y* = 11.65 ^∗^ *X*0.60	1.8	0.018	1.18E-02	1.25E-05
MAGIC	50	*Y* = 695.30 ^∗^ *X*-0.79	52.2	0.014	5.78E-04	7.99E-07	*Y* = 12.30 ^∗^ *X*0.57	2.4	0.022	2.52E-02	5.28E-05
MAGIC	100	*Y* = 960.18 ^∗^ *X*-0.85	56.1	0.011	1.79E-04	1.62E-07	*Y* = 8.97 ^∗^ *X*0.62	1.3	0.017	1.02E-02	8.70E-06
MAGIC	150	*Y* = 1,005.31 ^∗^ *X*-0.87	48.1	0.009	6.94E-05	5.65E-08	*Y* = 7.37 ^∗^ *X*0.65	0.9	0.015	5.75E-03	3.20E-06
MAGIC	200	*Y* = 1,186.84 ^∗^ *X*-0.90	40.2	0.006	1.29E-05	8.49E-09	*Y* = 6.50 ^∗^ *X*0.68	0.8	0.014	5.29E-03	2.45E-06

*The formula is *Y* = *m*^∗^*X*^*n*^. ^a^*X* is the number of markers; *Y* is the recombination bin size (Mb). ^b^A1 is the error for formula coefficient of *m*. ^c^A2 is the error for formula coefficient of *n*. ^d^*P*-value of formula coefficient *m*. ^e^*P*-value of formula coefficient *n*. ^f^*X* is the number of markers; Y is the recombination bin number. ^g^B1 is the error for formula coefficient of *m*. ^h^B2 is the error for formula coefficient of *n*. ^i^*P*-value of formula coefficient *m*. ^j^*P*-value of formula coefficient *n*.*

### Prediction of the Appropriate Population Size of Different Segregating Populations for Mapping

**FIGURE 5:**
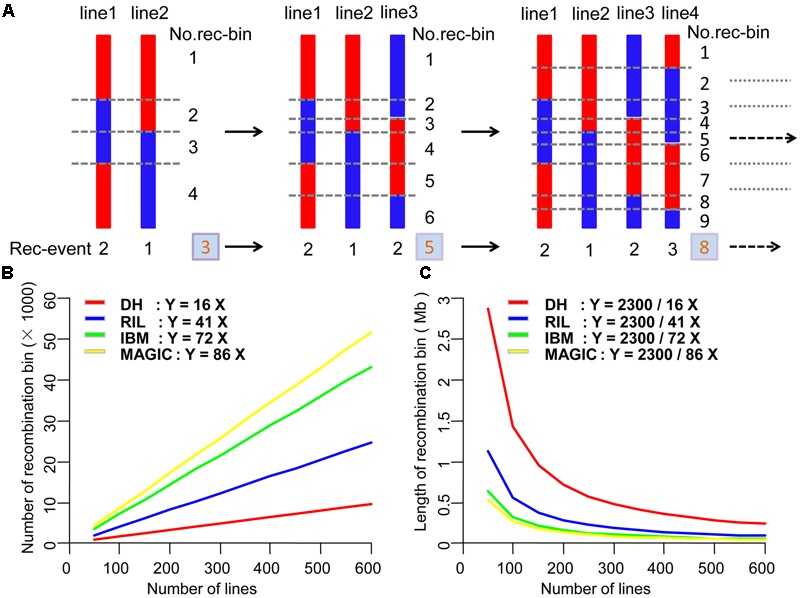
Estimation of theoretical number and size of recombination bins in four types of population. **(A)** Relationship between the number of recombination events and recombination bins. **(B)** Theoretical number of recombination bins in different numbers of DH, RIL, IBM, and MAGIC lines. **(C)** Theoretical length of recombination bins in different DH, RIL, IBM, and MAGIC lines. DH, double haploid; RIL, recombination inbred lines; IBM, intermated B73xMo17; MAGIC, multi-parent advanced generation inter-cross.

Across different types of segregating population, recombination bin number was correlated with recombination event number (**Figure 5A**); indeed, the total number of recombination events is equal to that of recombination bins. In the offspring of a population, recombination bin number was equal to the average number of recombination events per line multiplied by the number of lines (**Figure 5B**). The average number of recombination events was 16, 41, 72, and 86 in DH, RIL, IBM, and MAGIC populations, respectively. The length of the maize genome is about 2,300 Mb. Thus, the average size of recombination bin in one individual from each type of population was 2,300/16, 2,300/41, 2,300/72, and 2,300/86 Mb, respectively (**Figure 5C**). The maize genome comprises up to 40,000 genes (B73, Version 2 genome information). Therefore, a recombination bin of 57 Kb contains an average of one gene, although we note that there is large variation in this value across the genome. To reach this average bin size, with enough markers, the approximate number of lines should be 2,522, 984, 560, and 469 in DH, RIL, IBM, and MAGIC population, respectively.

### Comparing the Map Resolution for Different Types of Populations

In order to compare map resolution, 200 lines for each of the four population types were analyzed with 1,000 simulated phenotypes. For the 1,000 phenotypes, we mapped 269, 334, 357, and 370 QTLs for DH, RIL, IBM, and MAGIC populations, respectively, illustrating that with increased recombination, the number of QTLs mapped also increased. The average QTL likelihood interval was 9.96, 6.41, 3.25, and 1.87 Mb for DH, RIL, IBM, and MAGIC populations, respectively. The map resolution increase across different populations was significant (one-way ANOVA; *F* = 40.89; *P* < 2.0E-16; **Table 5**).

**Table 5:** Map resolution of different population types.

Pop.	Number of QTL	Mean ± sd (Mb)	Min (Mb)	Max (Mb)
DH	269	9.96 ± 8.75	0.59	60.93
RIL	334	6.41 ± 6.04	0.39	27.71
IBM	357	3.25 ± 2.98	0.07	20.31
MAGIC	370	1.87 ± 1.89	0.02	14.17

## Discussion

The output of molecular breeding can be significantly increased with the application of high-density markers and advancement in the genetic resources available for mapping. However, the precise number of markers for identifying specific characteristics of each population and mapping to the gene level is still a challenge. It is of prime importance to determine the optimal number of markers required for a mapping study, avoiding wasted efforts and inputs. Advancement in sequencing technology has significantly decreased the cost of developing high-density SNP markers but costly professional skills are required to handle huge datasets (Spindel et al., 2013). Furthermore, even a small mistake can cause misleading results and can waste time and resources. Therefore, we calculated the recombination characteristics, recombination bin number, and size to pick the optimal number of markers for a specific study.

Different high-density SNP chips developed in several plant species have been utilized in different ways to endeavor to resolve genomic problems (Ganal et al., 2011; Singh et al., 2015). Marker information is vital to explore the phenomenon of recombination and has permitted significant improvement in exploring the genetic characteristics of different populations (Pan et al., 2012). The SNP chip provided valuable resources for QTL mapping and may help to identify ideal sets of markers (Li X. et al., 2015). Our results indicated that the combination of 5,000 markers and 200 lines was optimal for mapping with high efficiency in DH, RIL, IBM, and MAGIC populations. Increasing the number of lines and markers can increase the map resolution but only by small increments. In previous studies, recombination break points could not be precisely inferred due to limited marker density. Clarifying details of the phenomena of recombination benefit greatly from high-density markers.

The process of gene cloning can be accelerated by using the recombination break points of different lines in one population instead of backcrossing and selfing to clone specific genes. This idea can save a lot of resources, accelerating progress in QTL manipulation and increasing the potentiality of maize and other crops. Furthermore, verifying results with different analytical approaches and case studies can accelerate implementation of genetic solutions to global challenges. The process of GWAS and its precision in QTL/gene mapping relies partly on marker density, because high-density markers can precisely delineate recombination break points (Bayer et al., 2015; Li X. et al., 2015). For marker-assisted breeding (MAS), the introgression of chromosomal segments delineated by high-density markers can help avoid “linkage drag” resulting from effects of other genes on a specific trait (Mammadov et al., 2012).

High-density markers can help to capture full sets of causal loci and precisely estimate allele effects in different types of mapping populations. Several mapping methods have been used for QTL/gene mapping with considerable success, all relying on balanced alleles to find the exact position of the concerned locus. Small numbers of recombination events only delimit loci to large intervals, while increasing marker density beyond a specific number wastes time and resources. If constraints exist on marker number and population size, one can choose among several types of segregating populations to achieve a specific goal. In this study, we set up formulas to help researchers estimate the map resolution available with proper marker density and line number for different types of populations with limited resources (**Table 4**). Our results showed that MAGIC populations, with the highest number of recombination events per line, offer higher map resolution than the other three population types studied.

Recombination frequency varied in different genomic regions, populations, and parents’ background. First, the recombination frequency varied in different chromosomal regions. In this study, we found that the recombination frequency was low in centromeric regions and high in telomere regions. The recombination frequency high regions correlated with high gene density, and phenotype will be affected by the high recombination frequency variance (Pan et al., 2016). This information could help researchers to accelerate fine mapping and gene cloning. For example, if you mapped one QTL/gene in high recombination frequency region, you could obtain more recombination lines than that in low recombination frequency region using the same size of population. Jointing the recombination lines and the corresponding phenotype, researchers could quickly clone the genes (Guan et al., 2017). Further, we found the recombination frequency varied in different populations. The MAGIC recombination frequency was highest, and the DH was the lowest. Except for the generation different, the parents’ number and background may be the reason for the recombination frequency variance. Based on this information we could get more different genetic background lines combining more recombination, which could help breeders to change the breeding strategy to obtain the ideal inbred lines. In this study, we also found that with different parents’ background, the offsprings’ recombination frequency varied, but the variance level was not beyond that of population type, which was also found in other study (Farkhari et al., 2011).

The total number of recombination events in each generation is relatively constant, while the locations of recombination break points are varied (Mirouze et al., 2012), helping us to utilize different strategies for construction of proper populations in order to improve map resolution (**Figure 1**). Recombination break points are not randomly distributed in the genome (Pan et al., 2016). Therefore, it is vital to identify the combination of line number and marker number that provides maximal resolution in the most suitable population type. Specific “recombination hotspots,” regions of chromosomes which experience more than average numbers of recombination events (Pan et al., 2016) could cause different physical resolution in different chromosomal regions, such as the 100 bins defined in the MaizeGDB website with 20 cM genetic length. Combining different populations can help to overcome this constraint, helping researchers to map genes quickly and accurately. Formulas to estimate the correlation between map resolution, marker number, and population size (**Table 4**) can help researchers to choose proper population size and genotyping strategy to facilitate fine mapping and cloning of genes. With improved low-cost sequencing technology, high-throughout genotyping is no longer a limitation and breeders can have high-density markers for populations under investigation. In the near future, the scientific community must solve how to combine population types, utilizing genome characteristics and population size together to perform mapping with maximal accuracy and minimal cost.

Breeding for desirable phenotypes mainly relies on combining new alleles and genes to generate and select ideotypes. The purpose of crossing is to bring forth new combinations of elite alleles in F_1_ populations (Wijnker and de Jong, 2008). Therefore, the base point for beneficial breeding is to pick out inbred lines and strive for more recombination events. Breeders construct different types of populations due to limitations of resources and time. Limited recombination events reduce the chances of obtaining elite allele combinations. We found that the average number of recombination events per line in the DH population was about 16, and the corresponding average length of chromosomal segments was 84.8 Mb, which was larger than that of RIL, IBM, and MAGIC. Therefore, with fewer recombination events, the recombinant chromosomal segments will be larger which will increase the level of difficulty to achieve more combinations. In this case, a large number of progeny lines will be required to identify desirable new phenotypes. Furthermore, we found that the average number of recombination events was as much as 72 and 41 per line in IBM and RIL, respectively. This difference was also clear in F_2_ and F_3_ generations. Recombination was further increased when inter-mating was performed in F_4_ and F_5_ generations (Liu H. et al., 2015). In the MAGIC population, lines were the outcome of multiple parents. This approach increased new allele combinations and allelic diversity levels in each offspring line. A line may not perform well in one generation but it may have the ability to receive desirable elite alleles. Therefore, with decreasing sequencing costs, the breeding community will be able to focus on many lines to achieve the target with maximum ease and efficiency. The prediction of phenotype with markers in each generation can enhance the efficiency of choosing breeding lines. In this study, we used different densities of markers and numbers of lines to construct formulas, which could predict how many lines could be used to produce allele combinations and find high efficiency breeding line prediction with a minimum of labor and time (**Table 4**). The idea to increase the level of recombination can be exploited by inter-mating F_6_ inbred lines with each other to improve recombination and help breeders to find elite lines. Sharing of experimental material among the scientific community facilitates discoveries such as recombination enhancement strategies that may help to meet the challenges of plant breeding and genetics.

## Author Contributions

QP designed this study. HG, FA, and QP performed the data analysis. HG, FA, and QP wrote the manuscript.

## Conflict of Interest Statement

The authors declare that the research was conducted in the absence of any commercial or financial relationships that could be construed as a potential conflict of interest.

## Footnotes

1 http://www.gramene.org/

2 http://www.maizego.org
