# Supplementary material for: Dissection of Recombination Attributes for Multiple Maize Populations Using a Common SNP Assay
Source: Front Plant Sci. 2017 Nov 30;8:2063. doi: 10.3389/fpls.2017.02063 (PMC5714861; doi:10.3389/fpls.2017.02063)
Supplement: Supplementary file 1 [file Table_1.PDF]

Supplementary Table S1 Summary of four types of population with markers and families

Pop type	No. pops	No. families[a]	No. polymorphic markers[b]	No. parents
DH	23	2,233	6,379-16,765	23
RIL	11	2,128	11,360-15,285	15
IBM	1	239	20,848	2
MAGIC	1	303	54,234	8

a The total family lines in each type of population, b The number of polymorphic markers in each type of population.
