# Supplementary material for: Dissection of Recombination Attributes for Multiple Maize Populations Using a Common SNP Assay
Source: Front Plant Sci. 2017 Nov 30;8:2063. doi: 10.3389/fpls.2017.02063 (PMC5714861; doi:10.3389/fpls.2017.02063)
Supplement: Supplementary file 2 [file Table_2.PDF]

Supplementary Table S2 Estimation of recombination bin number in four types of population with different sets of markers and families

No. markers	DH (50,100,150,200)				RIL (50,100,150,200)				IBM (50,100,150,200)				MAGIC (50,100,150,200)			
200	104	126	126	137	146	164	167	168	160	168	171	175	182	186	185	186
500	203	271	281	277	302	368	382	399	342	376	391	404	413	423	428	434
1,000	289	388	443	449	494	577	670	666	528	636	699	724	556	607	623	621
2,000	408	558	611	666	680	897	1,004	1,086	819	1,021	1,136	1,210	866	1,001	1,025	1,069
5,000	509	774	851	933	990	1,397	1,612	1,837	1,260	1,677	1,964	2,125	1,691	1,935	2,063	2,161
7,500	545	884	976	1,083	1,078	1,610	1,964	2,194	1,488	2,014	2,341	2,578	1,976	2,410	2,551	2,727
10,000	576	964	1,071	1,190	1,111	1,674	2,081	2,312	1,604	2,238	2,670	2,933	2,137	2,689	2,967	3,179
