# Supplementary material for: Dissection of Recombination Attributes for Multiple Maize Populations Using a Common SNP Assay
Source: Front Plant Sci. 2017 Nov 30;8:2063. doi: 10.3389/fpls.2017.02063 (PMC5714861; doi:10.3389/fpls.2017.02063)
Supplement: Supplementary file 3 [file Table_3.PDF]

Supplementary Table S3 Comparison of recombination bin number among different sets of lines with 7 sets of markers in four types of population

Pop type	100/50	150/100	200/150
DH	1.44	1.08	1.07
RIL	1.32	1.14	1.07
IBM	1.24	1.11	1.06
MAGIC	1.13	1.04	1.04
