# Supplementary material for: Dissection of Recombination Attributes for Multiple Maize Populations Using a Common SNP Assay
Source: Front Plant Sci. 2017 Nov 30;8:2063. doi: 10.3389/fpls.2017.02063 (PMC5714861; doi:10.3389/fpls.2017.02063)
Supplement: Supplementary file 4 [file Table_4.PDF]

Supplementary Table S4 Estimation of recombination bin size in four types of population with different sets of markers and lines

No. markers	DH (50,100,150,200)(Mb)				RIL (50,100,150,200)(Mb)				IBM (50,100,150,200)(Mb)				MAGIC (50,100,150,200)(Mb)			
200	17.25	14.81	14.81	13.42	12.83	11.19	11.48	11.33	11.57	11.06	11.13	10.63	10.62	10.43	10.22	10.13
500	9.45	7.25	6.95	7.05	6.53	5.4	5.19	4.98	5.74	5.2	5.01	4.84	4.87	4.67	4.59	4.36
1,000	6.83	5.12	4.82	4.44	4.06	3.48	2.99	3.02	3.78	3.14	2.87	2.77	2.83	2.49	2.39	2.35
2,000	4.9	3.6	3.2	3.02	2.97	2.26	2.02	1.87	2.46	1.97	1.78	1.67	1.8	1.48	1.41	1.29
5,000	3.94	2.61	2.21	2.17	2.05	1.46	1.27	1.11	1.61	1.21	1.03	0.95	0.96	0.79	0.72	0.63
7,500	3.2	2.2	1.8	1.6	1.89	1.27	1.04	0.93	1.37	1.01	0.87	0.79	0.8	0.62	0.56	0.47
10,000	2.8	1.8	1.4	1.2	1.7	1.13	0.91	0.82	1.27	0.91	0.76	0.69	0.73	0.55	0.48	0.39
