# Supplementary material for: Dissection of Recombination Attributes for Multiple Maize Populations Using a Common SNP Assay
Source: Front Plant Sci. 2017 Nov 30;8:2063. doi: 10.3389/fpls.2017.02063 (PMC5714861; doi:10.3389/fpls.2017.02063)
Supplement: Supplementary file 5 [file Table_5.PDF]

Supplementary Table S5 Comparison of recombination bin size among four types of population with different sets of markers and lines

No. families	Rate	Marker sets						
		200	500	1,000	2,000	5,000	7,500	10,000
	RIL/DH	0.74	0.69	0.60	0.61	0.52	0.59	0.61
	IBM/DH	0.67	0.61	0.55	0.50	0.41	0.43	0.45
50	MAGIC/DH	0.62	0.52	0.41	0.37	0.24	0.25	0.26
	RIL/DH	0.76	0.75	0.68	0.63	0.56	0.58	0.63
	IBM/DH	0.75	0.72	0.61	0.55	0.46	0.46	0.50
100	MAGIC/DH	0.70	0.64	0.49	0.41	0.30	0.28	0.31
	RIL/DH	0.78	0.75	0.62	0.63	0.57	0.58	0.65
	IBM/DH	0.75	0.72	0.60	0.56	0.47	0.48	0.54
150	MAGIC/DH	0.69	0.66	0.50	0.44	0.32	0.31	0.34
	RIL/DH	0.84	0.71	0.68	0.62	0.51	0.58	0.68
	IBM/DH	0.79	0.69	0.62	0.55	0.44	0.49	0.58
200	MAGIC/DH	0.76	0.62	0.53	0.43	0.29	0.30	0.33
