# Supplementary material for: Dissection of Recombination Attributes for Multiple Maize Populations Using a Common SNP Assay
Source: Front Plant Sci. 2017 Nov 30;8:2063. doi: 10.3389/fpls.2017.02063 (PMC5714861; doi:10.3389/fpls.2017.02063)
Supplement: Supplementary file 6 [file Image_1.PDF]

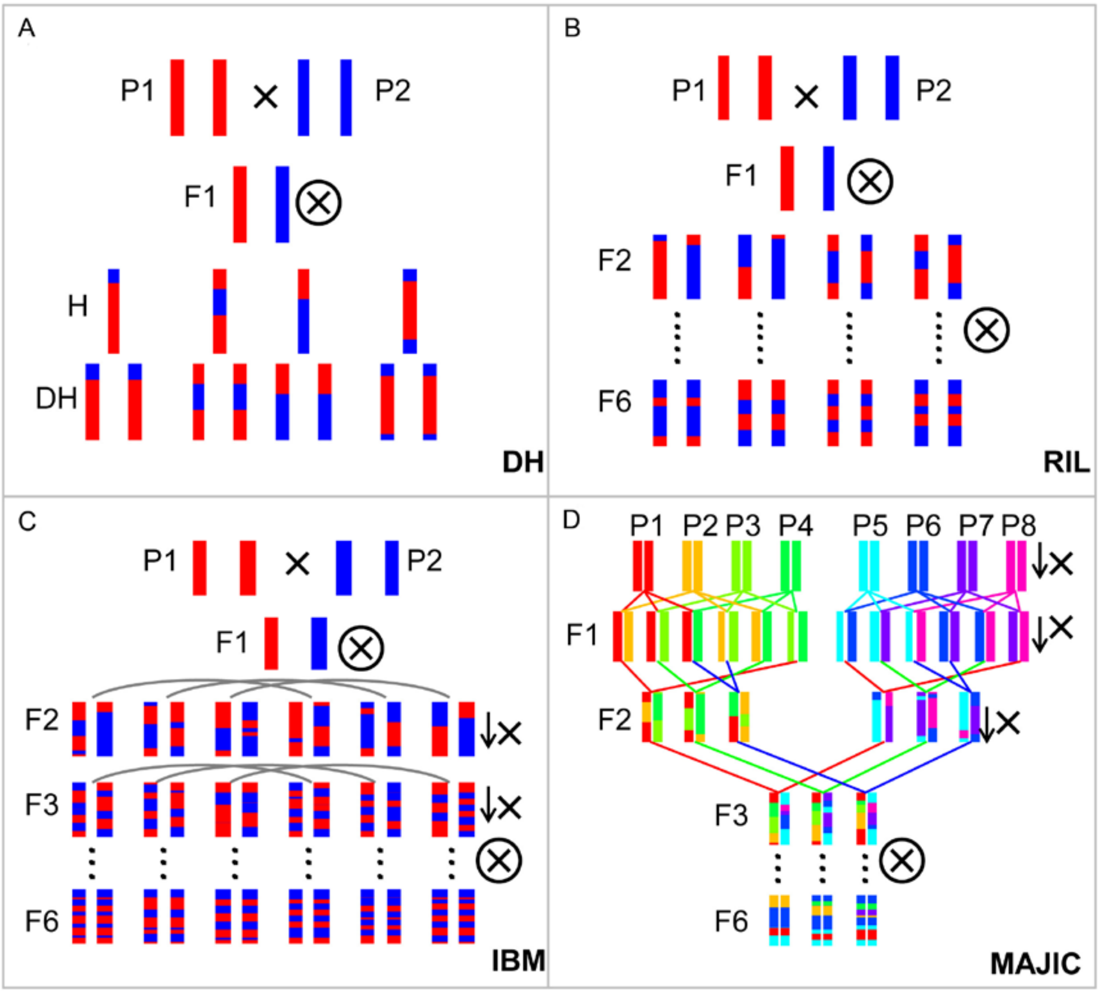

Supplementary Fig.S1 Summary of construction process of four types of segregating population (**A**), is the construction process of DH population. (**B**), is the construction process of RIL population. (**C**), is the construction process of IBM population. (**D**), is the construction process of eight parents MAGIC population.
