# Supplementary material for: Dissection of Recombination Attributes for Multiple Maize Populations Using a Common SNP Assay
Source: Front Plant Sci. 2017 Nov 30;8:2063. doi: 10.3389/fpls.2017.02063 (PMC5714861; doi:10.3389/fpls.2017.02063)
Supplement: Supplementary file 7 [file Image_2.PDF]

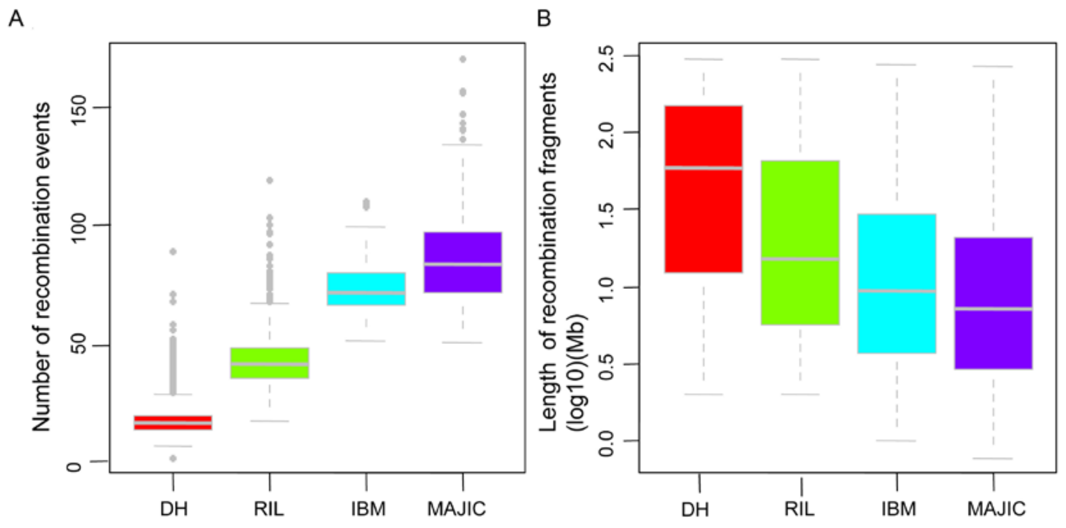

Supplementary Fig.S2 Distribution of recombination events number and chromosomal segment length in four types of segregating population. (**A**), is the distribution of recombination events number in four populations. The horizontal line in the box plots represents the median value of the recombination events number. The black "dots" in each box plot are outliers, the values of which are either higher than the sum of the third quantile value. (**B**), is the distribution of recombination segments size with log10 value in four populations. The horizontal line in the box plots represents the median value of the recombination segments length (log10).
